# The potential effects of untreated sleep-related breathing disorders on neuropathic pain, spasticity, and cardiovascular dysfunction following spinal cord injury: A cross-sectional prospective study protocol

**DOI:** 10.1371/journal.pone.0282860

**Published:** 2023-05-02

**Authors:** Julio Cesar Furlan, Eldon Loh, Mark Iskander Boulos

**Affiliations:** 1 Toronto Rehabilitation Institute, Lyndhurst Centre, University Health Network, Toronto, Ontario, Canada; 2 KITE Research Institute, University Health Network, Toronto, Ontario, Canada; 3 Department of Medicine, Division of Physical Medicine and Rehabilitation, University of Toronto, Toronto, Ontario, Canada; 4 Institute of Medical Science, University of Toronto, Toronto, Ontario, Canada; 5 Rehabilitation Sciences Institute, University of Toronto, Toronto, Ontario, Canada; 6 Institute of Health Policy, Management and Evaluation, University of Toronto, Toronto, Ontario, Canada; 7 Department of Physical Medicine and Rehabilitation, University of Western Ontario, London, ON, Canada; 8 Parkwood Institute Research, Lawson Health Research Institute, London, ON, Canada; 9 Sunnybrook Research Institute and Sunnybrook Health Sciences Centre, Hurvitz Brain Sciences Research Program, Toronto, ON, Canada; 10 Department of Medicine, Division of Neurology, University of Toronto, Toronto, ON, Canada; Public Library of Science, UNITED STATES

## Abstract

**Introduction:**

Sleep-related breathing disorders (SRBDs), neuropathic pain, spasticity and cardiovascular autonomic dysfunction are common after spinal cord injury (SCI). Prior studies suggest that systemic inflammation following SCI may be implicated in the development of neuropathic pain, spasticity and cardiovascular dysfunction. Given that SRBDs also cause a systemic inflammatory response, we hypothesized that individuals with SCI who develop more severe SRBDs would experience more intense neuropathic pain, more severe spasticity and more significant cardiovascular autonomic dysfunction.

**Methods:**

This cross-sectional prospective study will explore the previously understudied hypothesis that SRBDs are associated with increased neuropathic pain, spasticity, and cardiovascular autonomic dysfunction in adult individuals with low-cervical/high-thoracic (injury level at C5 to T6), complete/incomplete (ASIA Impairment Scale A, B, C or D) SCI.

**Discussion:**

To our knowledge, no prior study has addressed this clinically relevant question on whether the degree of SRBDs affects the intensity of neuropathic pain, spasticity, and cardiovascular autonomic dysfunction in individuals with SCI. We anticipate that the results of this original study will provide key information for a future clinical trial on the use of continuous positive airway pressure (CPAP) therapy for moderate-to-severe SRBDs, which may better control neuropathic pain, spasticity, and cardiovascular autonomic dysfunction among individuals with SCI.

**Trial registration:**

The research protocol for this study was registered in the ClinicalTrials.gov website (NCT05687097). https://clinicaltrials.gov/ct2/show/NCT05687097.

## Introduction

Spinal cord injury (SCI) is a potentially catastrophic event for individuals who sustain vary degrees of motor, sensory, and autonomic deficits and for society due to the related economic burden. The worldwide incidence of traumatic SCI varies from 6.2 to 174 per million inhabitants yearly and its global prevalence varies from 50 to 906 per million inhabitants [1]. A recent review underscored the paucity of studies on the epidemiology of non-traumatic SCI, but reported its incidence to vary between 6 and 68 per million inhabitants and its prevalence varied from 1,120 to 2,310 per million inhabitants in a few studies [2]. Although SCI has a relatively modest incidence, its burden is substantial. In Canada, the estimated lifetime costs per individual with SCI vary from $1.2 million USD for incomplete paraplegia to $2.4 million USD for complete tetraplegia [3]. Secondary medical conditions following SCI can affect many body systems, impact health, and limit function, quality of life, and societal participation [4,5]. In Australia, the total direct costs of visits to emergency departments and hospital readmissions for treatment of secondary complications within a 2-year period after SCI were estimated to be $65,250 USD and $4.1 million USD, respectively [4].

Cardiovascular autonomic dysfunction, neuropathic pain, spasticity, and sleep disorders (particularly SRBDs) are among the most common and costly secondary medical conditions following SCI [4]. Individuals with tetraplegia often face significant cardiovascular autonomic dysfunction, including low baseline blood pressure (44% to 51%), orthostatic hypotension (59% to 74%), autonomic dysreflexia (26% to 90%), and cardiac arrhythmias (e.g. bradycardia: 17% to 77%) [5–10]. Neuropathic pain reportedly occurs in 48% to 92% of those with SCI, and spasticity is found in 40% to 60% of individuals living with tetraplegia [5,6,11]. While SCI has been linked to an increased frequency of obstructive and central sleep apnea among those with tetraplegia (48% to 91%, and 63%, respectively) and those with paraplegia (63% and 13%, respectively), the underlying mechanisms of SRBD after SCI are poorly understood [12,13]. Overall, post-SCI SRBDs seem to reflect a complex interplay among several factors including increased body mass, reduced lung volumes altering “traction forces” and airway patency, preference for sleeping in the supine position, an altered balance of parasympathetic and sympathetic tone in the airways, neuroplastic changes in the medullary respiratory control circuitry, altered chemosensitivity of respiratory motor output, and medication-induced effects [13].

Interestingly, prior investigations reported that some pathophysiological mechanisms are common between SRBDs and other secondary medical conditions such as cardiovascular autonomic dysfunction, neuropathic pain, and spasticity following SCI. For instance, SCI prompts systemic inflammation and neuroinflammatory processes associated with the production and release of proinflammatory cytokines [14,15]. While the neuronal circuits involved in neuropathic pain and spasticity seem to be at least partially distinct, the proposed pathophysiological mechanisms of both neuropathic pain and spasticity involve disinhibition from loss of descending pathways or interneurons, neuronal hyperexcitability, ectopic firing, sprouting, receptor upregulation, deafferentation effects on rostral or caudal neurons, glial activation, and neuroinflammation [16–18]. More specifically, there is a growing body of evidence suggesting that neuroinflammation persists into the chronic stage after SCI, similar to neuropathic pain and spasticity [15,16,19,20]. A neuroinflammatory response, mainly in the microglia and astrocytes, has been linked to hyperactivity in spinal pain pathways during SCI-induced pain [19,20]. Also, astrocyte activation is a major constituent of neuroinflammation, and persistent activation of astrocytes contributes to chronic SCI-induced pain [20]. Systemic inflammation and neuroinflammation also occur in non-disabled individuals with moderate-to-severe SRBDs [21,22]. In a meta-analysis, the levels of systemic inflammatory markers (including C-reactive protein [CRP], Tumor Necrosis Factor alpha [TNF-α], interleukins 6 and 8) were found to be higher in non-disabled patients with SRBDs compared to control subjects [21]. Moreover, this effect was proportional to the severity of SRBDs [21].

SCI can also result in autonomic dysfunction through the interruption of descending inhibitory pathways on cervico-thoracic and sacral preganglionic neurons, which may cause cardiovascular, urinary, sexual, and/or gastrointestinal complications [9,20]. Individuals with SCI are predisposed to sympathetic overactivity due to the loss of supraspinal control. The interactions between the autonomic nervous system and pain modulation are quite complex. Plastic reorganization of spinal autonomic circuitry caudal to the level of SCI enhances the sympathetic anti-inflammatory reflex [20,23]. Conversely, sympathetic stimulation can promote pro-inflammatory responses involving immune cells, resulting in the release of inflammatory mediators (including pain-related cytokines) [20,24,25]. Catecholaminergic and sympathetic alterations play a key role in the pathophysiology of cardiovascular disorders related to SRBDs in non-disabled people [26]. It is still unclear how individuals with SCI respond to sympathetic and catecholaminergic challenges from hypoxia during apneas and hypopneas.

Overall, SRBDs, cardiovascular autonomic dysfunction, neuropathic pain, and spasticity are common complications after SCI that likely have interacting effects [5–10]. Yet, the potential clinical relationships between SRBDs and other secondary medical conditions such as cardiovascular autonomic dysfunction, neuropathic pain, and spasticity in individuals with SCI have been under-studied [12]. Accordingly, we hypothesized that SRBDs would be associated with increased neuropathic pain, spasticity, and cardiovascular autonomic dysfunction in adult individuals with low-cervical-to-high-thoracic (injury level at C5 to T6), complete or incomplete SCI.

## Participants and methods

This cross-sectional prospective study will explore the previously understudied hypothesis that SRBDs are associated with increased neuropathic pain, worsened spasticity, and more severe cardiovascular autonomic dysfunction in adult individuals with low-cervical-to-high-thoracic (injury level at C5 to T6), complete or incomplete (ASIA Impairment Scale A, B, C or D), subacute or chronic (at least 1 month since injury) SCI. The research protocol for this study has been approved and renewed by the Research Ethics Board of the University Health Network (Coordinated Approval Process for Clinical Research [CAPCR] ID# 19–5285) since May 7, 2020.

This study will examine the following hypotheses:

1. Individuals with SCI and untreated SRBDs will develop more intense neuropathic pain;
2. Individuals with SCI and untreated SRBDs will develop more severe spasticity;
3. Individuals with SCI and untreated SRBDs will develop more significant cardiovascular dysfunction (i.e. reduced heart rate variation, more frequent episodes of autonomic dysreflexia during sleep).

The primary objectives of this study are: (a) to evaluate the potential association of neuropathic pain with SRBDs in individuals with SCI; (b) to assess the potential association between spasticity and SRBDs in individuals with SCI; and (c) to study the potential relationship between cardiovascular autonomic dysfunction and SRBDs in individuals living with SCI.

The secondary objectives of this study are: (i) to confirm that a home-based or hospital-unattended sleep study is a feasible alternative for the diagnosis of SRBDs in individuals with tetraplegia or paraplegia; and (ii) to compare the costs of a home-based sleep study with conventional in-laboratory polysomnography using cost-minimization analysis.

### Definitions and diagnosis

The International Association for the Study of Pain defines neuropathic pain as “pain caused by a lesion or disease of the somatosensory nervous system [27]”. This definition was also adopted in the recent CanPain SCI Clinical Practical Guidelines [11].

According to Lance, “spasticity is a motor disorder characterized by a velocity-dependent increase in tonic stretch reflexes (muscle tone) with exaggerated tendon jerks, resulting from hyperexcitability of the stretch reflex, as one component of the upper motor neuron syndrome [28]”.

Obstructive, central, and mixed sleep apnea are defined according to diagnostic criteria from the American Academy of Sleep Medicine [29,30]. For the purpose of this study, SRBD will be defined as an apnea–hypopnea index (AHI) ≥ 5 events per hour of sleep. According to its severity, sleep apnea is classified as mild if 5 ≤ AHI <15 events, moderate if 15 ≤ AHI ≤ 30 events, and severe if AHI > 30 events [31]. While polysomnography is the gold standard for diagnosing SRBDs, ambulatory home-based or hospital-unattended sleep tests (using the ResMed ApneaLink Air^TM^ device) can be used as a practical, less expensive, validated, and reliable surrogate for diagnosis of the SRBDs [32–34].

Episodes of autonomic dysreflexia will be identified when there is a sudden increase in the systolic blood pressure (>20% from baseline) accompanied by altered heart rate after an episode of apnea or hypopnea identified by the ambulatory sleep test usingthe ResMed ApneaLink Air^TM^ device [7,35,36]. Participants with complete disruption of the descending vasomotor autonomic pathways typically have a significant reduction in their heart rate, and even bradycardia, during episodes of autonomic dysreflexia [35–37]. In contrast, participants with incomplete disruption of the descending vasomotor autonomic pathways may respond to an increase in their systolic blood pressure with a concomitant increase in heart rate, and even tachycardia, during episodes of autonomic dysreflexia [35–37].

### Outcome measures

The primary outcome measures of this study will include:

1. The degree of neuropathic pain using the Visual Analog Scale (VAS), which is a self-reported scale with a range from 0 (no pain) to 10 (“worst pain”) [38]. In addition, the name and quantity of any analgesic that each participant is taking will be recorded at the time of the assessment of the degree of neuropathic pain.
2. The degree of spasticity in all four extremities assessed using the Modified Ashworth Scale (MAS), which is a clinician-based assessment that consists of a 5-point nominal scale using subjective clinical assessments of tone varying from 0 –‘no increases in tone’ to 4 –‘limb rigid in flexion or extension (abduction/ adduction)’ [39]. Furthermore, the name and quantity of any muscle relaxant that each participant is taking will be recorded at the time of assessment of the degree of spasticity.
3. The mean, minimum and maximum systolic and diastolic blood pressures, and heart rate during sleep.
4. The mean, minimum and maximum R-R interval during sleep.
5. The number of episodes of autonomic dysreflexia preceded by an event of apnea or hypopnea.

Secondary outcome measures will include:

- Feasibility of a home-based or hospital-unattended test based on the experience of the participants when using the ResMed ApneaLink Air^TM^ device;
- Economic analysis comparing costs of a home-based or hospital-unattended sleep test with in-laboratory polysomnography for the diagnosis of SRBDs in individuals living with SCI.
- Clinical assessment of the participants’ sleep as assessed using the STOP-Bang questionnaire (including questions on Snoring, Tiredness, Observed stoppages in breathing or choking/gasping, high blood Pressure, Body mass index > 35 kg/m^2^, Age older than 50 years, Neck size large, Gender = male). Of note, a STOP-Bang score < 3 suggests no significant symptoms or signs of SRBDs, whereas a higher STOP-Bang score is associated with a greater degree of SRBD [40–43].
- Serum CRP level will be analyzed as a surrogate for probable systemic inflammatory response after ruling out infectious and other causes of inflammation.

### Research plan

**Fig 1:**
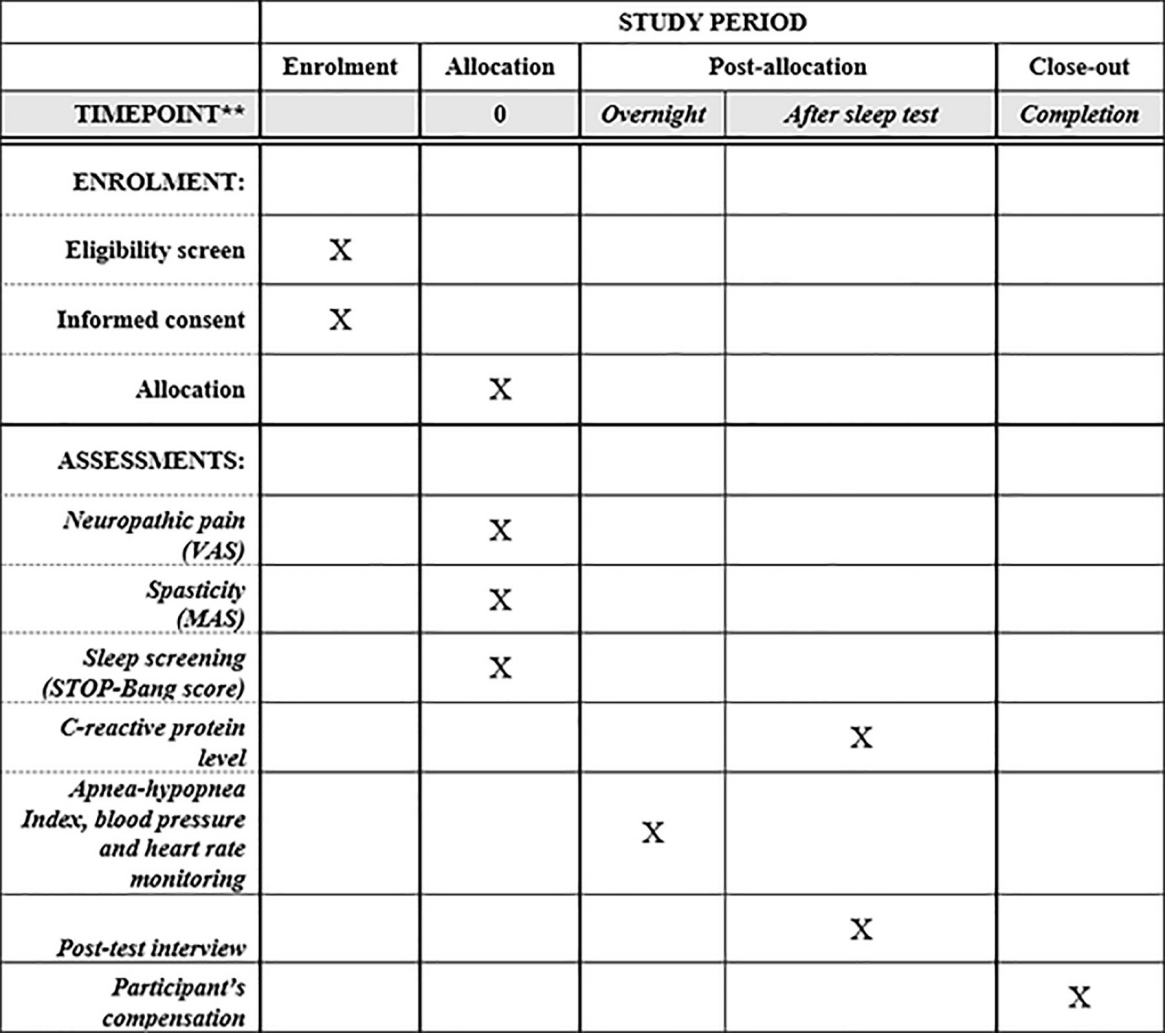
Standard Protocol Items: Recommendations for Interventional Trials (SPIRIT) template on the protocol for this research project.

This research study will enroll outpatients and inpatients from the largest, tertiary spinal cord rehabilitation center in Canada. The research protocol for this study is summarized in **Fig 1**. Potential participants will be identified by any clinician working in the outpatient services. Also, an institutional central recruitment service will be contracted to identify potential participants who are admitted to the rehabilitation center. The institutional central recruitment service is a mandatory service that checks on every inpatient regarding their interest in participating in any research project during their hospital stay. A study research assistant at the rehabilitation center will invite potential outpatient and inpatient participants for this research project. Only English-speaking, adult participants who are capable of providing written consent to participate in the study will be included. If the participant provides verbal consent to participate in the study, but cannot sign the consent form due to a disability, one of their relatives can sign the consent form with the participant present as a witness.

Potential participants who signed the consent form will be assessed to confirm their eligibility, check for warning signs for SRBDs (e.g., fragmented and unrefreshing sleep, daytime sleepiness, snoring and/or gasping for air during sleep) and check their STOP-Bang scores. In addition, those enrolled into the study will be assessed by the study research assistant for neuropathic pain using the VAS, as well as assessed by a board-certified neurologist for spasticity using the MAS.

Afterwards, each study participant will undergo concomitant overnight assessments using a home-based or hospital-unattended sleep test (i.e., ResMed ApneaLink Air^TM^ device), cardiovascular monitoring that includes beat-to-beat continuous systolic and diastolic blood pressure and heart rate recording (using a CareTaker^TM^ device), and continuous electrocardiographic recording (using a Bittium Faros 180^TM^ device).

### Data collection

Baseline data will be collected for each participant, which will include individual’s sex, age at SCI onset, time since the injury to study enrolment, neurological level of SCI, severity of SCI as assessed using the AIS, body mass index, neck circumference, and Modified Mallampati classification (based on the “relative position of the palate and base of tongue inside the mouth—Class I: all the oropharynx including tonsils, pillars, soft palate, and the tip of uvula can be easily visible—Class II: tonsils’ upper polo and uvula are visible—Class III: part of the uvula and soft palate are visible—Class IV: just the hard palate and part of soft palate are visible”) [44], and presence or absence of nasal, throat or palate anatomic variants that contribute to SRBDs (*e*.*g*., retrognathia, macroglossia, tonsillar hypertrophy, elongated/ enlarged uvula, or high arched/narrow hard palate) [45].

In addition, data on first-line pharmacological treatments (and their daily doses) used to manage spasticity (e.g., baclofen, tizanidine, benzodiazepines) and neuropathic pain (e.g., gabapentoids, tricyclic antidepressants, opioids) during the study assessments will be collected.

For the purpose of studying the feasibility of the home-based or hospital-unattended test, the number of times a participant is required to repeat the overnight screening sleep test until successful completion of the screening sleep test, which means that at least 4 hours of acceptable quality data are collected. Additionally, each participant will be invited for a 1-hour, semi-structured interview that will allow them to share their experience of undergoing a home-based or hospital-unattended test.

For the purpose of economic analyses, the costs of the home-based or hospital-unattended sleep test will be compared to the costs of an in-laboratory overnight sleep test (i.e., conventional polysomnogram). The costs of the home-based or hospital-unattended test will include the expenses of device depreciation based on the market price for a ResMed ApneaLink Air^TM^ device and supplies for the test. Personnel salaries to instruct participants on the use of the home-based or hospital-unattended test (as applicable), provide overnight remote support (for the home-based sleep test), or to instruct, apply and remove the device (for the hospital-unattended test) will also be recorded. The costs of the polysomnogram will be obtained from a hospital-based sleep clinic located in Toronto. The professional and technical fees for interpretation and reporting the results of the sleep studies will be derived from the Ontario Health Insurance Plan (OHIP)–Schedule of Benefits, Physician Services. All costs will be converted and adjusted to the USD at the final year of the study.

### Data analysis

The baseline characteristics among the four study groups will be analyzed using the Kruskal-Wallis test (if non-parametric data) or ANOVA (if parametric data). Study groups will also be compared using Fisher exact test with regard to categorical variables.

The association of the degree of SRBD as assessed using AHI with each outcome measure (i.e. VAS, MAS, systolic and diastolic blood pressure during sleep, heart rate during sleep, R-R interval during sleep, the number of episodes of autonomic dysreflexia during sleep, and serum CRP level) will be tested using univariate and multivariate data analysis. First, Pearson correlation coefficient analyses will be used to compare the AHI with each outcome measure. Second, ANOVA with *post hoc* Bonferroni correction will be used to compare the outcomes measures among four study groups as follow: (i) Individuals without SRBDs after SCI; (ii) Individuals with mild SRBRs after SCI; (iii) Individuals with moderate SRBRs after SCI; and (iv) Individuals with severe SRBRs after SCI. The latter will allow the research team to check if there is an association of the AHI with each outcome measure any particular study group(s).

Third, multiple regression models will be used to test the association between the AHI and each outcome measure after adjusting for major potential confounders (including individual’s sex, age at the SCI onset, time since the SCI, level and severity of SCI, and doses of the first-line pharmacological treatments for either neuropathic pain or spasticity when applied). Finally, sensitivity analyses will be performed to test the robustness of the results from the above data analyses. For the sensitivity analyses, the original VAS and MAS will be adjusted by adding the corresponding minimal clinically important difference to the original VAS and MAS scores. Therefore, study participants on a first-line pharmacological treatment for neuropathic pain will have 2.5 points (the minimal clinically important difference for VAS) added to their original VAS to estimate the presumed VAS without treatment (adjusted-VAS) [46]. Similarly, study participants taking a first-line pharmacological treatment for spasticity will have 1 point (the minimal clinically important difference for MAS) added to their original MAS to estimate the presumed MAS without treatment (adjusted-MAS) [47]. Further, sensitivity analyses for MAS will be performed for the total MAS (i.e., the overall MAS score after summing up the MAS in each extremity), and the highest MAS score in any extremity. Notably, the MAS score of “1+” will be converted to 1.5 for the purpose of the data analyses.

Data analyses on the practicality of a home-based or hospital-unattended test will include a descriptive analysis of the number of times a participant was required to repeat the overnight screening sleep test in order to successfully complete it. Also, qualitative analysis will be carried out using the participants’ responses to a questionnaire about their experience when undergoing a home-based or hospital-unattended test.

The costs of the home-based or hospital-unattended test will be compared with the costs of the in-laboratory overnight sleep test (i.e., conventional polysomnogram) using a cost-minimization analysis. This approach for the cost effectiveness analysis will be undertaken because the “effectiveness” of the polysomnogram is considered similar to the home-based or hospital-unattended test [32–34]. All the costs will be converted and adjusted to the USD at the final year of the study.

### Sample size and study feasibility

A sample size of 70 individuals with SCI was estimated to provide a minimum power of 80% and a significance of 5% for every statistical method above described, including the multiple regression analysis with up to 7 independent variables (i.e., outcome measure of interest, individual’s sex, age at the SCI onset, level and severity of SCI, and use of up to two medications for management of neuropathic pain or spasticity).

According to the publically available data from the National SCI Statistical Center, 40% of individuals with SCI have an AIS A/B/C/D SCI at C5 to T6 [48]. For the purpose of feasibility analysis, the leading rehabilitation center in this study admits 300 patients with SCI every year of which 120 patients sustain an AIS A/B/C/D SCI at C5 to T6 [48]. Of those, approximately 60 patients will develop SRBDs [12]. When considering a conservative screening-to-recruitment ratio of 2:1 for cohort studies, we anticipate completing this study within the grant period [49].

## Discussion

Current literature indicates that SRBDs, cardiovascular autonomic dysfunction, neuropathic pain, and spasticity are common secondary medical conditions following SCI, which likely have interacting effects [5–10]. The proposed research study will examine the under-studied, potential clinical relationships between SRBDs and other secondary medical conditions such as neuropathic pain, spasticity, and cardiovascular autonomic dysfunction in individuals living with low-cervical/high-thoracic, subacute/chronic SCI [12].

### Unattended-hospital or home-based sleep apnea tests

Individuals living with SCI who have symptoms suggestive of SRBDs commonly face substantial challenges when undergoing a polysomnogram due to the lack of financial support for a personal support worker at night or the lack of accessibility (e.g., bed, washroom) in the sleep disorders clinic. Given this, the use an unattended-hospital or home-based sleep apnea test (using ResMed ApneaLink Air^TM^ device) is a putatively practical, less costly, validated and reliable surrogate for diagnosis of the SRBDs [32–34]. The results of a prior single-center prospective observational study demonstrated that the ApneaLink Plus ambulatory sleep monitoring was a feasible and practical diagnostic strategy for screening people for SRBDs after stroke in their home or hospital, without the need for an overnight stay in a specialized sleep clinic [34]. The use of an unattended-hospital or a home-based sleep apnea test can be a feasible and practical diagnostic strategy to increase the feasibility and acceptability of screening for SRBDs among individuals with SCI and, hence, may increase the frequency of the diagnosis of SRBDs. However, individuals living with a disability due to SCI may experience particular challenges when undergoing an unattended-hospital or home-based sleep apnea test, which led us to include user support and evaluation by a trained research assistant to help every study participant when undergoing an unattended-hospital or a home-based sleep apnea test. Moreover, the experience and perceptions of the participants will be complied and analyzed in a qualitative study that aims to determine the major barriers and limitations of the use of an unattended-hospital or a home-based sleep test. Overall, this research project will provide key information on how to improve the diagnosis of SRBDs among individuals living with SCI who are at higher risk for developing SRBDs, but have particular challenges related to their degree of impairment and disability.

Despite the potential advantages of the unattended-hospital or home-based sleep apnea test, this method actually underestimates the AHI. The reason is that the ratio used to estimate the AHI has the number of apneas and hypopneas recorded during sleep by the device as the numerator, and the time since the device is turned on until it is turned off on the denominator. Given that the ResMed ApneaLink Air^TM^ device does not record the electroencephalogram, which is used to identify the stages of sleep during conventional in-laboratory polysomnography, the total number of apneas and hypopneas is divided by an inflated period of time when the individual is still awake for a short period of time before falling asleep and for a short period of time between awakening and stopping the recording. In the protocol for this research project, the participants will be asked to estimate the time when they fell asleep during the sleep study and how long it took them to stop the recording after awakening. This information will then be used to adjust the period of time during sleep.

### Outcome measures

The instruments for assessment of the degree of neuropathic pain (i.e., VAS) and spasticity (i.e., MAS) in this study are considered reliable, responsive and valid for their purpose in the SCI population. However, the use of pharmacological treatments for neuropathic pain and spasticity will mitigate the strength of association between AHI and these two secondary medical conditions. As it is not reasonable and ethical to ask study participants to refrain from taking their regular medications for management of neuropathic pain and spasticity, the protocol for data analysis in this study includes a series of sensitivity analyses using data after adjustments for the estimated drug effects based on the minimal clinically important differences for the VAS and MAS. As mentioned above, 2.5 points (the minimal clinically important difference for VAS) will be added to the original VAS in those study participants who are taking a first-line pharmacological therapy for neuropathic pain in order to estimate the presumed VAS without treatment (adjusted-VAS) [46]. Similarly, study participants will have 1 point (the minimal clinically important difference for MAS) added to their original MAS in those study participants who are taking a first-line pharmacological therapy for spasticity in order to estimate the presumed MAS without treatment (adjusted-MAS) [47]. The principle behind those adjusted outcome measures is that the participants are still taking their medications because they are at least partially effective in the control of neuropathic pain and spasticity at the level of the minimal clinically important differences.

Similar to what was discussed above regarding the sleep testing, the cardiovascular monitoring records will include a period of time before the participant falls asleep and a period of time when they are awake but the recordings from the CareTaker^TM^ and Bittium Faros 180^TM^ devices were not stopped yet. Participants will then be asked to estimate the time when they fell asleep during the sleep study and how long it took for them to stop the recording. This information will be used to disregard those periods of time that either precede or follow sleep from the cardiovascular monitoring records.

Another potential pitfall related to cardiovascular monitoring records is the risk of overestimation of apnea/hypopnea-induced episodes of autonomic dysreflexia during sleep by inadvertently including episodes of autonomic dysreflexia due to other causes. The latter will be identified using the participant’s report and/or checking their medical files (if they are hospitalized), when there was a trigger of autonomic dysreflexia during sleep (e.g. a participant wakes up for an intermittent catheterization for neurogenic bladder, or for a bowel movement associated with neurogenic bowel). For the purpose of this research project, only the apnea/hypopnea-induced episodes of autonomic dysreflexia during sleep will be considered to test the study hypothesis.

Finally, the participants will be asked to have their blood drawn for serum CRP level to screen for systemic inflammatory response. As there will be inpatients and outpatients participating in this study, all the laboratory blood tests will be carried out by a single company that provides high-quality services using standardized assays and methods across Canada. This will facilitate the comparisons and interpretation of the results from this research project.

## Supporting information

S1 Checklist SPIRIT-Outcomes 2022 checklist (for combined completion of SPIRIT 2013 and SPIRIT-Outcomes 2022 items)^a^. (PDF)

S1 File (PDF)

## References

[1] Furlan JC, Tator CH. Global epidemiology of traumatic spinal cord injury. In: Morganti-Kossman C, Raghupathi R, Maas A, editors. Traumatic brain and spinal cord injury: Challenges and Developments. First edition ed. Cambridge: Cambridge University Press; 2012. p. 360.

[2] New PW, Cripps RA, Bonne Lee B. Global maps of non-traumatic spinal cord injury epidemiology: towards a living data repository. Spinal Cord. 2014;52(2):97–109. doi: 10.1038/sc.2012.165 . 23318556

[3] Krueger H, Noonan VK, Trenaman LM, Joshi P, Rivers CS. The economic burden of traumatic spinal cord injury in Canada. Chronic Dis Inj Can. 2013;33(3):113–22. . 23735450

[4] Gabbe BJ, Nunn A. Profile and costs of secondary conditions resulting in emergency department presentations and readmission to hospital following traumatic spinal cord injury. Injury. 2016;47(8):1847–55. doi: 10.1016/j.injury.2016.06.012 . 27343134

[5] Noreau L, Noonan VK, Cobb J, Leblond J, Dumont FS. Spinal cord injury community survey: a national, comprehensive study to portray the lives of canadians with spinal cord injury. Top Spinal Cord Inj Rehabil. 2014;20(4):249–64. doi: 10.1310/sci2004-249 ; PubMed Central PMCID: PMC4252126. 25477739 PMC4252126

[6] Noreau L, Proulx P, Gagnon L, Drolet M, Laramee MT. Secondary impairments after spinal cord injury: a population-based study. Am J Phys Med Rehabil. 2000;79(6):526–35. doi: 10.1097/00002060-200011000-00009 . 11083303

[7] Krassioukov AV, Furlan JC, Fehlings MG. Autonomic dysreflexia in acute spinal cord injury: an under-recognized clinical entity. J Neurotrauma. 2003;20(8):707–16. Epub 2003/09/11. doi: 10.1089/089771503767869944 . 12965050

[8] Furlan JC, Fehlings MG, Shannon P, Norenberg MD, Krassioukov AV. Descending vasomotor pathways in humans: correlation between axonal preservation and cardiovascular dysfunction after spinal cord injury. J Neurotrauma. 2003;20(12):1351–63. doi: 10.1089/089771503322686148 . 14748983

[9] Furlan JC, Fehlings MG. Cardiovascular complications after acute spinal cord injury: pathophysiology, diagnosis, and management. Neurosurg Focus. 2008;25(5):E13. doi: 10.3171/FOC.2008.25.11.E13 . 18980473

[10] Hector SM, Biering-Sorensen T, Krassioukov A, Biering-Sorensen F. Cardiac arrhythmias associated with spinal cord injury. J Spinal Cord Med. 2013;36(6):591–9. doi: 10.1179/2045772313Y.0000000114 ; PubMed Central PMCID: PMC3831320. 24090076 PMC3831320

[11] Loh E, Guy SD, Mehta S, Moulin DE, Bryce TN, Middleton JW, et al. The CanPain SCI Clinical Practice Guidelines for Rehabilitation Management of Neuropathic Pain after Spinal Cord: introduction, methodology and recommendation overview. Spinal Cord. 2016;54 Suppl 1:S1–6. doi: 10.1038/sc.2016.88 . 27444714

[12] Chiodo AE, Sitrin RG, Bauman KA. Sleep disordered breathing in spinal cord injury: A systematic review. J Spinal Cord Med. 2016;39(4):374–82. Epub 2016/04/15. doi: 10.1080/10790268.2015.1126449 ; PubMed Central PMCID: PMC5102283. 27077573 PMC5102283

[13] Fuller DD, Lee KZ, Tester NJ. The impact of spinal cord injury on breathing during sleep. Respir Physiol Neurobiol. 2013;188(3):344–54. doi: 10.1016/j.resp.2013.06.009 ; PubMed Central PMCID: PMC4017769. 23791824 PMC4017769

[14] Furlan JC, Krassioukov AV, Fehlings MG. Hematologic abnormalities within the first week after acute isolated traumatic cervical spinal cord injury: a case-control cohort study. Spine (Phila Pa 1976). 2006;31(23):2674–83. Epub 2006/11/02. doi: 10.1097/01.brs.0000244569.91204.01 . 17077735

[15] Schwab JM, Zhang Y, Kopp MA, Brommer B, Popovich PG. The paradox of chronic neuroinflammation, systemic immune suppression, autoimmunity after traumatic chronic spinal cord injury. Exp Neurol. 2014;258:121–9. Epub 2014/07/16. doi: 10.1016/j.expneurol.2014.04.023 ; PubMed Central PMCID: PMC4099970. 25017893 PMC4099970

[16] Finnerup NB. Neuropathic pain and spasticity: intricate consequences of spinal cord injury. Spinal Cord. 2017;55(12):1046–50. Epub 2017/07/12. doi: 10.1038/sc.2017.70 . 28695904

[17] Tashiro S, Shinozaki M, Mukaino M, Renault-Mihara F, Toyama Y, Liu M, et al. BDNF Induced by Treadmill Training Contributes to the Suppression of Spasticity and Allodynia After Spinal Cord Injury via Upregulation of KCC2. Neurorehabil Neural Repair. 2015;29(7):677–89. Epub 2014/12/21. doi: 10.1177/1545968314562110 . 25527489

[18] Corleto JA, Bravo-Hernandez M, Kamizato K, Kakinohana O, Santucci C, Navarro MR, et al. Thoracic 9 Spinal Transection-Induced Model of Muscle Spasticity in the Rat: A Systematic Electrophysiological and Histopathological Characterization. PLoS One. 2015;10(12):e0144642. Epub 2015/12/30. doi: 10.1371/journal.pone.0144642 ; PubMed Central PMCID: PMC4705098. 26713446 PMC4705098

[19] Walters ET. Neuroinflammatory contributions to pain after SCI: roles for central glial mechanisms and nociceptor-mediated host defense. Exp Neurol. 2014;258:48–61. Epub 2014/07/16. doi: 10.1016/j.expneurol.2014.02.001 . 25017887

[20] Walters ET. How is chronic pain related to sympathetic dysfunction and autonomic dysreflexia following spinal cord injury? Auton Neurosci. 2018;209:79–89. Epub 2017/02/06. doi: 10.1016/j.autneu.2017.01.006 ; PubMed Central PMCID: PMC5529270. 28161248 PMC5529270

[21] Nadeem R, Molnar J, Madbouly EM, Nida M, Aggarwal S, Sajid H, et al. Serum inflammatory markers in obstructive sleep apnea: a meta-analysis. J Clin Sleep Med. 2013;9(10):1003–12. Epub 2013/10/16. doi: 10.5664/jcsm.3070 ; PubMed Central PMCID: PMC3778171. 24127144 PMC3778171

[22] Sapin E, Peyron C, Roche F, Gay N, Carcenac C, Savasta M, et al. Chronic Intermittent Hypoxia Induces Chronic Low-Grade Neuroinflammation in the Dorsal Hippocampus of Mice. Sleep. 2015;38(10):1537–46. Epub 2015/06/19. doi: 10.5665/sleep.5042 ; PubMed Central PMCID: PMC4576327. 26085297 PMC4576327

[23] Ueno M, Ueno-Nakamura Y, Niehaus J, Popovich PG, Yoshida Y. Silencing spinal interneurons inhibits immune suppressive autonomic reflexes caused by spinal cord injury. Nat Neurosci. 2016;19(6):784–7. Epub 2016/04/19. doi: 10.1038/nn.4289 ; PubMed Central PMCID: PMC4882232. 27089020 PMC4882232

[24] Kingery WS. Role of neuropeptide, cytokine, and growth factor signaling in complex regional pain syndrome. Pain Med. 2010;11(8):1239–50. Epub 2010/08/14. doi: 10.1111/j.1526-4637.2010.00913.x . 20704672

[25] Tse KH, Chow KB, Leung WK, Wong YH, Wise H. Primary sensory neurons regulate Toll-like receptor-4-dependent activity of glial cells in dorsal root ganglia. Neuroscience. 2014;279:10–22. Epub 2014/08/31. doi: 10.1016/j.neuroscience.2014.08.033 . 25171787

[26] Bisogni V, Pengo MF, Maiolino G, Rossi GP. The sympathetic nervous system and catecholamines metabolism in obstructive sleep apnoea. J Thorac Dis. 2016;8(2):243–54. doi: 10.3978/j.issn.2072-1439.2015.11.14 ; PubMed Central PMCID: PMC4739957. 26904265 PMC4739957

[27] Davidoff G, Roth E, Guarracini M, Sliwa J, Yarkony G. Function-limiting dysesthetic pain syndrome among traumatic spinal cord injury patients: a cross-sectional study. Pain. 1987;29(1):39–48. Epub 1987/04/01. doi: 10.1016/0304-3959(87)90176-X . 3588000

[28] Lance JW. Symposium synopsis. In: Feldman RG, Young RR, Koella WP, editors. Spasticity: Disordered Control. Chicago: Yearbook Medical; 1980. p. 485–94.

[29] Berry RB, Budhiraja R, Gottlieb DJ, Gozal D, Iber C, Kapur VK, et al. Rules for scoring respiratory events in sleep: update of the 2007 AASM Manual for the Scoring of Sleep and Associated Events. Deliberations of the Sleep Apnea Definitions Task Force of the American Academy of Sleep Medicine. J Clin Sleep Med. 2012;8(5):597–619. doi: 10.5664/jcsm.2172 ; PubMed Central PMCID: PMC3459210. 23066376 PMC3459210

[30] Iber C, Ancoli-Israel S, Chesson A, Quan SF. The AASM Manual for the Scoring of Sleep and Associated Events: Rules, Terminology and Technical Specifications. 1st ed ed. Westchester, IL 2007.

[31] Epstein LJ, Kristo D, Strollo PJ Jr., Friedman N, Malhotra A, Patil SP, et al. Clinical guideline for the evaluation, management and long-term care of obstructive sleep apnea in adults. J Clin Sleep Med. 2009;5(3):263–76. ; PubMed Central PMCID: PMC2699173. 19960649 PMC2699173

[32] Nigro CA, Dibur E, Malnis S, Grandval S, Nogueira F. Validation of ApneaLink Ox for the diagnosis of obstructive sleep apnea. Sleep Breath. 2013;17(1):259–66. doi: 10.1007/s11325-012-0684-4 . 22447171

[33] Erman MK, Stewart D, Einhorn D, Gordon N, Casal E. Validation of the ApneaLink for the screening of sleep apnea: a novel and simple single-channel recording device. J Clin Sleep Med. 2007;3(4):387–92. ; PubMed Central PMCID: PMC1978315. 17694728 PMC1978315

[34] Boulos MI, Elias S, Wan A, Im J, Frankul F, Atalla M, et al. Unattended Hospital and Home Sleep Apnea Testing Following Cerebrovascular Events. J Stroke Cerebrovasc Dis. 2017;26(1):143–9. Epub 2016/10/09. doi: 10.1016/j.jstrokecerebrovasdis.2016.09.001 . 27717683

[35] Furlan JC, Fehlings MG. Cardiovascular complications after acute spinal cord injury: pathophysiology, diagnosis, and management. Neurosurgical focus. 2008;25(5):E13. Epub 2008/11/05. doi: 10.3171/FOC.2008.25.11.E13 . 18980473

[36] Furlan JC. Autonomic dysreflexia: a clinical emergency. J Trauma Acute Care Surg. 2013;75(3):496–500. doi: 10.1097/TA.0b013e31829fda0a . 24089120

[37] Furlan JC, Fehlings MG, Shannon P, Norenberg MD, Krassioukov AV. Descending vasomotor pathways in humans: correlation between axonal preservation and cardiovascular dysfunction after spinal cord injury. Journal of neurotrauma. 2003;20(12):1351–63. Epub 2004/01/30. doi: 10.1089/089771503322686148 . 14748983

[38] Furlan JC, Noonan V, Singh A, Fehlings MG. Assessment of impairment in patients with acute traumatic spinal cord injury: a systematic review of the literature. J Neurotrauma. 2011;28(8):1445–77. Epub 2009/12/25. doi: 10.1089/neu.2009.1152 ; PubMed Central PMCID: PMC3143408. 20030559 PMC3143408

[39] Noonan V, Zhu J, Mak J, Querée M, Chi J. Ashworth and Modified Ashworth Scale (MAS) Vanciuver: University of British Columbia; 2019 [cited 2021 12/06/2021]. Available from: https://scireproject.com/wp-content/uploads/Clinician-Summary_Ashworth-v.7.0.pdf.

[40] Miller JN, Kupzyk KA, Zimmerman L, Pozehl B, Schulz P, Romberger D, et al. Comparisons of measures used to screen for obstructive sleep apnea in patients referred to a sleep clinic. Sleep Med. 2018;51:15–21. Epub 2018/08/06. doi: 10.1016/j.sleep.2018.06.007 . 30077956

[41] Chiu HY, Chen PY, Chuang LP, Chen NH, Tu YK, Hsieh YJ, et al. Diagnostic accuracy of the Berlin questionnaire, STOP-BANG, STOP, and Epworth sleepiness scale in detecting obstructive sleep apnea: A bivariate meta-analysis. Sleep Med Rev. 2017;36:57–70. Epub 2016/12/07. doi: 10.1016/j.smrv.2016.10.004 . 27919588

[42] Tan A, Yin JD, Tan LW, van Dam RM, Cheung YY, Lee CH. Predicting obstructive sleep apnea using the STOP-Bang questionnaire in the general population. Sleep Med. 2016;27–28:66–71. Epub 2016/12/13. doi: 10.1016/j.sleep.2016.06.034 . 27938922

[43] Panchasara B, Poots AJ, Davies G. Are the Epworth Sleepiness Scale and Stop-Bang model effective at predicting the severity of obstructive sleep apnoea (OSA); in particular OSA requiring treatment? Eur Arch Otorhinolaryngol. 2017;274(12):4233–9. Epub 2017/09/01. doi: 10.1007/s00405-017-4725-2 . 28856422

[44] Zonato AI, Martinho FL, Bittencourt LR, de Oliveira Campones Brasil O, Gregorio LC, Tufik S. Head and neck physical examination: comparison between nonapneic and obstructive sleep apnea patients. Laryngoscope. 2005;115(6):1030–4. Epub 2005/06/04. doi: 10.1097/01.MLG.0000163494.19965.DC . 15933515

[45] Shelgikar AV, Chervin R. Approach to and evaluation of sleep disorders. Continuum (Minneap Minn). 2013;19(1 Sleep Disorders):32–49. doi: 10.1212/01.CON.0000427214.00092.0f . 23385693

[46] Han ZA, Song DH, Oh HM, Chung ME. Botulinum toxin type A for neuropathic pain in patients with spinal cord injury. Ann Neurol. 2016;79(4):569–78. doi: 10.1002/ana.24605 ; PubMed Central PMCID: PMC4825405. 26814620 PMC4825405

[47] Shaw L, Rodgers H, Price C, van Wijck F, Shackley P, Steen N, et al. BoTULS: a multicentre randomised controlled trial to evaluate the clinical effectiveness and cost-effectiveness of treating upper limb spasticity due to stroke with botulinum toxin type A. Health Technol Assess. 2010;14(26):1–113, iii-iv. Epub 2010/06/03. doi: 10.3310/hta14260 . 20515600

[48] 2017 Annual Statistical Report–Complete Public Version [Internet]. University of Alabama at Birmingham. 2017 [cited 2018]. Available from: https://www.nscisc.uab.edu/Public_Pages/ReportsStats.

[49] Craven BC, Balioussis C, Hitzig SL, Moore C, Verrier MC, Giangregorio LM, et al. Use of screening to recruitment ratios as a tool for planning and implementing spinal cord injury rehabilitation research. Spinal Cord. 2014;52(10):764–8. Epub 2014/08/20. doi: 10.1038/sc.2014.126 . 25135057

