# Supplementary material for: The potential effects of untreated sleep-related breathing disorders on neuropathic pain, spasticity, and cardiovascular dysfunction following spinal cord injury: A cross-sectional prospective study protocol
Source: PLoS One. 2023 May 2;18(5):e0282860. doi: 10.1371/journal.pone.0282860 (PMC10153696; doi:10.1371/journal.pone.0282860)
Supplement: S1 File — (PDF) [file pone.0282860.s002.pdf]

ETHICS PROTOCOL SUBMISSION

Untreated sleep-related breathing disorders as an aggravating factor for neuropathic pain, cardiovascular dysfunction and spasticity following spinal cord injury

Principal Investigator: Dr. Julio Furlan

Toronto Rehabilitation Institute
University Health Network

Protocol Version #8
Date 21/06/2021

Sponsor: Ontario Neurotrauma Foundation & Ministry of Health of Ontario

TABLE OF CONTENTS

List of Abbreviations 3
Protocol Summary 3
1. General Information 4
1.1. Protocol title and date. 4
2. Background and rationale 4
2.1. Summary of previous findings 5
2.2. Rationale for the study 6
2.3. Potential Risks 9
2.4. Potential Benefits 10
2.5. Description of the population to be studied. 11
2.5. Compliance with protocol, ICH GCP and regulatory requirements 12
3.2. Study hypothesis. 12
4. Study Design 12
4.1. Primary and secondary endpoints 12
4.2. Trial intervention 12
4.3. The timeline of the study 13
5. Selection of Participants 16
5.1. Inclusion criteria. 16
5.2. Exclusion and non-inclusion criteria. 16
5.3. Withdrawal of Participants 16
7. Statistics 17
10. Ethics 18
10.2. Consent process 18
10.3. Review and approval of the study by the REB 18
10.4. Review and approval of any amendment. 18
11. Data Handling and Record Keeping 18
12. Financing and Insurance 19
13. Publication Policy 19
14. References 20

List of Abbreviations

ASIA: American Spinal Injury Association (author of the ISNCSCI)
CPAP-Continuous positive airway pressure
CRF-Case report form
ISCOS: International Spinal Cord Society
REB: Research ethical board
SAE-A serious adverse event
SCI: Spinal cord injury
SRBDs: Sleep-related breathing disorders
VAS-Visual analog scale

Protocol Summary

Title
Untreated sleep-related breathing disorders as an aggravating factor for neuropathic pain, cardiovascular dysfunction and spasticity following spinal cord injury.

Short title
Sleep and secondary complications of SCI.

Type and phase of the study
Cross-sectional, prospective study.

Sample Size
N= 70 participants

Study Population
Cervical/thoracic (injury level at C1 to T6), complete/incomplete (AIS A, B, C or D) SCI.

Accrual Period
5-years

Study Duration
May 1st 2019 to March 30th 2024 (36 months)

Study Intervention
Home-based or hospital-unattended sleep test (using ResMed ApneaLink Air^{TM}) can be used as a practical, less expensive, validated and reliable surrogate for diagnosis of the SRBDs.

Primary Objective
The linkage of untreated sleep-related breathing disorders (SRBDs) with neuropathic pain, cardiovascular dysfunction, and spasticity after SCI.

Exploratory Objectives
Increase awareness of the SRBDs in the SCI population and among healthcare rehab professionals.

General Information

1.1. Protocol title and date.
Untreated sleep-related breathing disorders as an aggravating factor for neuropathic pain following spinal cord injury.

Protocol Version #7, March 23, 2021

Untreated sleep-related breathing disorders as an aggravating factor for neuropathic pain, cardiovascular dysfunction and spasticity following spinal cord injury

1.2. Name and title of the investigators

Study Principal Investigator
Dr. Julio Cesar Furlan
Lyndhurst Center, Toronto Rehab
520 Sutherland Drive
Toronto, Ontario, M4G 3V9, Canada
Phone:+1-416-597-3422; Fax:+1-416-425-9923
E-mail: julio.furlan@uhn.ca

Study Co- Investigators:
Dr. Eldon Loh MD, FRCPC
Phone: 519-685-4080
Email: Eldon.Loh@sjhc.london.on.ca

Dr. Mark I. Boulos MD, FRCPC
Phone: 416-480-4473
Email: mark.boulos@sunnybrook.ca

Dr. David Berlowitz (PhD)
Phone: 011 (61) 06-9496-53-90
Email: djberl@unimelb.edu.au

2.0 Background and Rationale

2.1. Summary of previous findings

The majority of individuals living with SCI experience chronic pain, including nociceptive and neuropathic pain.[1] Nociceptive pain is caused by activation of the normal peripheral terminals of primary sensory neurons (“nociceptors”) that detect signs of actual or incipient tissue injury such as inflammation.[2] Neuropathic pain is caused by injury or disease of the nervous system.[3] SCI-induced neuropathic pain is considered a pathological, maladaptive consequence of damage to, and inflammation of, the central nervous system.[4, 5] Neuropathic pain occurs in 48% to 92% for individuals with SCI.[6-8] Given that neuropathic pain is a frequent and usually permanent complication that is typically resistant to treatment, there is a great need for more effective approaches to manage SCI-induced neuropathic pain.[9]

Sleep disorders after SCI

A variety of sleep disorders have been described in individuals with SCI including insomnia and sleep-related breathing disorders (SRBDs). In a review of 11 epidemiological studies, the mean prevalence of SRBDs was 6% in men and 4% in women in the general population.[10] In comparison, SRBDs have been reported in 48% to 91% of individuals with tetraplegia, and 13% to 63% of individuals with paraplegia.[11, 12] Although the frequency of SRBDs after SCI is much greater than in the able-bodied population, this is still a largely under-studied secondary condition in the SCI population.[11]

Untreated sleep-related breathing disorders as an aggravating factor for neuropathic pain, cardiovascular dysfunction and spasticity following spinal cord injury

In a recent qualitative study, 20 individuals with SCI voiced several barriers to good quality sleep including SCI-related dysfunction, care, sleep environment, pain, anxiety, and avoidance.[13] In summary, “sleep problems, such as those described by participants in this study, have the potential to exacerbate several conditions that commonly occur after SCI and to significantly affect health-related quality of life.”

Pain and sleep disorders
Pain has a negative impact on sleep, and SRBDs can modulate changes in pain processing and sensitivity.[8] In addition, several psychosocial factors (e.g., depression, anxiety) may exacerbate pain-related distress and disability in individuals with SCI, as has been reported in able-bodied people with SRBDs.[14] Therefore, a bidirectional relationship between pain and sleep disorders in the SCI population is plausible, even though this clinically important association has not been properly addressed in the literature. Overall, at least two pathophysiological mechanisms are common to SCI-induced neuropathic pain and SRBDs: neuroinflammation and autonomic disturbances.

Overall, neuropathic pain, SRBDs and cardiovascular autonomic dysfunction are common complications after SCI that likely have interplay effects.[6, 7, 15-18] However, the potential relationship between SRBDs and other secondary complications such as neuropathic pain and cardiovascular autonomic dysfunction in individuals with SCI have not been previously studied.[11]

Spasticity and sleep disorders
Similar to neuropathic pain, spasticity is a common secondary complication after SCI that has been reported 40% to 60% of the individuals living with tetraplegia.[1-3]It is still unclear how spinal cord injured people with an underlying sympathetic overactivity due to the loss of supraspinal control respond to those sympathetic and catecholaminergic changes during and after hypoxia. Also, the effects of sleep-related hypoxia on the sympathetically-denervated muscles of individuals with SCI remain unknown. Prior studies revealed an increased muscle sympathetic nerve activity under hypoxic conditions, which persisted even after removal of acute short-term exposure to intermittent hypoxia.[4] While a previous study showed that muscle oxygenation is not significantly affected by intermittent hypoxia exposure, the prefrontal cortex suffered detrimental effects of hypoxia in the prefrontal cortex in able-bodied individuals.[5] One may speculate that anterior horn cells may also be vulnerable to hypoxia and, hence, the response of the low motor neurons caudal to SCI may overreact (or underreact) to hypoxia and sympathetic drive. Of note, a recent systematic review on the physiologic and psychologic triggers for spasticity exacerbation found no previous study on the effects of SRBDs on the spasticity in individuals with SCI.[6] Nonetheless, the potential effects of untreated SRBDs on the degree of spasticity after SCI have never been properly examined.

2.2 Rationale

Impact of outcomes on stakeholders
It is anticipated that this original research project will confirm an association of untreated SRBDs with neuropathic pain, cardiovascular dysfunction and spasticity after SCI, which will have an impact on stakeholders as follows:

Untreated sleep-related breathing disorders as an aggravating factor for neuropathic pain, cardiovascular dysfunction and spasticity following spinal cord injury

(i) For individuals living with SCI, proper management of SRBDs could have multiple important benefits. While this study will specifically explore reductions in neuropathic pain, cardiovascular dysfunction and spasticity, which would significantly improve quality of life for those with SCI, other important benefits of SBRDs including reductions in daytime sleepiness, fatigue, mood disorder, cognitive effects) could be achieved. This may facilitate adherence to management of SRBDs which, according to the current standard of care, includes continuous positive airway pressure (CPAP) therapy for those individuals with SCI and moderate-to-severe SRBD. In this group of patients, CPAP therapy may become a non-pharmacological option for management of neuropathic pain and cardiovascular dysfunction when moderate-to-severe SRBD co-exists.

(ii) Health care professionals will potentially add another non-pharmacological option (i.e., CPAP therapy) for management of neuropathic pain, cardiovascular dysfunction, and spasticity in those individuals living with SCI who are diagnosed with a moderate-to-severe SRBD. In addition, screening for SRBDs among individuals with SCI will likely improve due to the multiple benefits of the CPAP therapy.

(iii) Administrators of rehabilitation facilities and policymakers will likely be encouraged to offer home-based or hospital-unattended sleep studies in the rehabilitation facilities that care for individuals living with SCI. Home-based or hospital-unattended sleep studies could be a more cost-effective strategy that favors early diagnosis and treatment of SRBDs, which may save hospital and healthcare costs.

Appropriate engagement of stakeholders

In prior meetings with individuals living with SCI and representative consumers working at SCI Ontario, Ms. Nancy Xia and Mr. Peter Athanasopoulos confirmed their commitment to collaborate with us in this research project to refine data collection, interpretation and dissemination. Furthermore, healthcare professionals at Lyndhurst Centre were very engaged in the initial discussions about the potential outcomes of this study including the use of home-based or hospital-unattended sleep study, and the benefits of the CPAP therapy on neuropathic pain, cardiovascular dysfunction and spasticity after SCI. The investigators obtained full support for this research project.

Likelihood for knowledge uptake by target audience

The potential benefits of this study have been briefly discussed with several patients with SCI, health care professionals, and administrators at Lyndhurst Centre. Overall, the comments were overwhelmingly supportive for this research project with particular reference to the benefits of using home-based or hospital unattended devices for the sleep studies in the SCI population. The possibility of CPAP therapy to concomitantly treat moderate-to-severe SRBD and improve neuropathic pain, cardiovascular dysfunction and spasticity is very appealing to individuals with living SCI.

Scientific

Neuropathic pain, cardiovascular dysfunction, spasticity and SRBDs are common and clinically relevant secondary complications after SCI. According to the literature, at least two pathophysiological mechanisms are common to SCI-induced neuropathic pain and SRBDs: neuroinflammation and autonomic disturbances.

Untreated sleep-related breathing disorders as an aggravating factor for neuropathic pain, cardiovascular dysfunction and spasticity following spinal cord injury

SCI prompts systemic inflammation and neuroinflammation mechanisms associated with the production and release of proinflammatory cytokines.[19, 20] There is a growing body of evidence which suggests that neuroinflammation perpetuates into the chronic stage after SCI, similar to neuropathic pain.[2, 20, 21] A neuroinflammatory response, mainly in the microglia and astrocytes, has been linked to hyperactivity in spinal pain pathways during SCI-induced pain.[2, 21] Also, astrocyte activation is a major constituent of neuroinflammation, and persistent activation of astrocytes contributes to chronic SCI-induced pain.[2] Systematic inflammation and neuroinflammation also occur in able-bodied individuals with moderate-to-severe SRBDs.[22, 2]3 In a meta-analysis, the levels of systemic inflammatory markers (including CRP, TNF-α, interleukins 6 and 8) were found to be higher in able-bodied patients with SRBDs compared to control subjects.[22] Moreover, this effect was proportional to the severity of SRBDs.[22]

SCI can also result in autonomic dysfunction through the interruption of descending inhibitory pathways on cervico-thoracic and sacral preganglionic neurons (causing cardiovascular, urinary, sexual, and gastrointestinal complications).[2, 17] Individuals with SCI are predisposed to sympathetic overactivity due to the loss of supraspinal control. The interactions between autonomic nervous system and pain modulation are quite complex. Plastic reorganization of spinal autonomic circuitry caudal to the level of SCI enhance sympathetic anti-inflammatory reflex.[2, 24] Conversely, sympathetic stimulation can promote pro-inflammatory responses involving immune cells and release inflammatory mediators (including pain-related cytokines).[2, 25, 26] Catecholaminergic and sympathetic alterations play a key role in the pathophysiology of cardiovascular disorders related to SRBDs in able-bodied people.[27] It is still unclear how individuals with SCI respond to sympathetic and catecholaminergic challenges from hypoxia.

Prior studies revealed an increased muscle sympathetic nerve activity under hypoxic conditions, which persisted even after removal of acute short-term exposure to intermittent hypoxia.[4] While a previous study showed that muscle oxygenation is not significantly affected by intermittent hypoxia exposure, the prefrontal cortex suffered detrimental effects of hypoxia in the prefrontal cortex in able-bodied individuals.[5] One may speculate that anterior horn cells may also be vulnerable to hypoxia and, hence, the response of the low motor neurons caudal to SCI may overreact (or underreact) to hypoxia and sympathetic drive. Of note, a recent systematic review on the physiologic and psychologic triggers for spasticity exacerbation found no previous study on the effects of SRBDs on the spasticity in individuals with SCI.[6] Nonetheless, the potential effects of untreated SRBDs on the degree of spasticity after SCI have never been properly examined.

Overall, neuropathic pain, cardiovascular autonomic dysfunction, spasticity and SRBDs are common complications after SCI that likely have interplay effects.[6, 7, 15-18] However, the potential relationship between SRBDs and other secondary complications such as neuropathic pain and cardiovascular autonomic dysfunction in individuals with SCI have not been previously studied.[11] By comparing SCI individuals without and with different degrees of SRBDs, this original study will fulfil the current knowledge gap in the literature and provide key information for a future clinical trial on the effects of CPAP therapy for better control of neuropathic pain and cardiovascular dysfunction in individuals living with SCI and SRBDs.

2.2.1 Investigational Team

This research project is a joined endeavor of an experienced team with expertise on Medical Research, Spinal Cord Medicine, Neurorehabilitation, Neurology and Sleep Medicine that will be led by:

- ***Julio C. Furlan*** (MD, LLB, MBA, PhD, MSc, FRCPC) is a staff neurologist and clinician investigator at Lyndhurst Centre, Toronto Rehabilitation Institute, University Health Network, with subspecialty in NeuroRehabilitation and Neural Repair as well as Clinical Neurophysiology, and expertise in clinical epidemiology, and health economics. He is the principal investigator of this research project. He will dedicate 20% of his time for the research project.
- ***Eldon Loh*** (MD, FRCPC) is a staff physiatrist and Medical Director of the Spinal Cord Injury Program at Parkwood Hospital, a physiatrist and interventionalist at the St. Joseph's Pain Clinic, and an Associate Professor, Department of Physical Medicine and Rehabilitation at Western University. Dr. Loh has led the most recently published CanPain SCI Clinical Practice Guidelines on management of neuropathic pain in the SCI population. He is a co-investigator of this research project. He will dedicate 10% of his time for the research project.
- ***Mark I. Boulos*** (BSc, MSc, MD, FRCPC) is a staff neurologist in the Division of Neurology, Department of Medicine, Sunnybrook Health Sciences Centre, an affiliate scientist at the Sunnybrook Research Institute, and an Assistant Professor, Division of Neurology, Department of Medicine, University of Toronto, Ontario, Canada. In addition to his expertise in Sleep Medicine, Dr. Boulos has experience in the use of home-based testing (using ResMed ApneaLink Air™) in the diagnosis of sleep disorders among patients with neurological diseases (especially, stroke). He is a co-investigator of this research project. He will dedicate 10% of his time for the project.
- ***David Berlowitz*** (PhD) David Berlowitz is a physiotherapist with the Victorian Respiratory Support Service who holds The University of Melbourne Chair in Physiotherapy at Austin Health. Dr. Berlowitz was obtained a PhD degree in 2004, in which he discovered that acute cervical SCI results in sudden and severe obstructive sleep apnea. He is a consultant for this research project.

As previously mentioned, the project team also includes Ms. Nancy Xia and Mr. Peter Athanasopoulos, who bring their experience as consumers and research collaborators. Ms. Xia has been actively assisting in the knowledge transfer of the CanPain SCI Clinical Practice Guidelines for management of neuropathic pain after SCI (see attached her letter of support). Mr. Peter Athanasopoulos has been leading the Ontario SCI Solutions Alliance for better access to the best practice care for all individuals living with SCI. Mr. Athanasopoulos will help us to recruit participants for the research project if necessary and, more importantly, he will enrich the knowledge dissemination of the study results with the objective of increasing awareness and facilitating access to early diagnosis and proper management of SRBDs, which can potentially improve SCI-induced neuropathic pain and cardiovascular dysfunction (see attached his letter of support from the SCI Ontario).

2.3 Potential Risks and Benefits

2.3.1 Potential Risk

- **Risk to the participant**

a) There may be some minor discomfort from wearing the ApneaLink Sleep Breathing Monitor.
b) There may be some minor disturbance of participant's usual sleep on the night that she/he is wearing the ApneaLink device.
c) Participants may learn something about their sleep that they were unaware of, such as their probability of having a sleep disorder, which could cause some anxiety or discomfort. Those participants who are diagnosed with moderate-to-severe SRBD are expected to try on and adhere to CPAP therapy. If the participant refuse to be adherent to CPAP therapy, the healthcare professional caring for them is required by law to report the diagnosis to the Ministry of Transportation of Ontario that likely will suspend the participant's driving privileges.
e) Blood collections can sometimes cause bruising, pain, or very rarely, loss of consciousness.
f) Participants may learn something about their health related to abnormal blood test that they were unaware of, which may require further investigations and treatment by the participants' family physician; this could cause some anxiety or discomfort.

- **Risks to the community/public**
  To the best of our knowledge: none.

- **Risks to the institution**
  To the best of our knowledge: none**.**

2.3.2 Potential Benefits

- Benefits to the participant
  The results of this study could help thousands of individuals living with SCI who experience neuropathic pain, cardiovascular dysfunction and SRBDs. We anticipate that, by confirming the association of neuropathic pain and cardiovascular dysfunction with SRBDs, this study will increase awareness of the early diagnosis and properly management of the SRBDs among individuals living with SCI. Also, a home-based sleep test is presumably a more feasible diagnostic tool for SRBDs in the SCI population than the conventional polysomnography that requires overnight stay in a sleep disorder clinic.

- Benefits to the community/public
  The knowledge-to-action framework including two interacting phases (i.e., knowledge creation and action cycle) as described by Graham et al. will be adopted in this project.[7] During knowledge creation, the knowledge producers should tailor their activities to the needs of potential users. During the action cycle, implementers and users are ought to work together for the success of the knowledge dissemination with focus on change healthcare practice and individuals' behaviors.
  As proposed by Dr. Heather Gainforth and endorsed by the Ontario Neurotrauma Foundation, an integrated knowledge transfer approach will be undertaken in this research project that has established a partnership with the SCI community from the initial steps to dissemination of the results. This innovative initiative enhances development of research projects that are meaningful for the consumers (i.e., individuals living with SCI) and other stakeholders. This approach presumably facilitates the dissemination of the study findings,

knowledge acquisition and, eventually, best practice implementation. We are thrilled that Ms. Nancy Xia and Mr. Peter Athanasopoulos have agreed to collaborate with us in this exciting and innovative research project. Ms. Xia has been actively assisting in the knowledge transfer of the CanPain SCI Clinical Practice Guidelines for management of neuropathic pain after SCI. Ms. Xia's own experience as an individual living with SCI and neuropathic pain will also be a great asset in the conduction and dissemination of this research outcomes (see attached her letter of support). Mr. Peter Athanasopoulos has been leading the Ontario SCI Solutions Alliance for better access to best practice care for all individuals living with SCI. Mr. Athanasopoulos will help us to recruit participants for the research project if necessary and, more importantly, he will enrich the knowledge dissemination of the study results with the objective of increasing awareness and facilitating access to early diagnosis and proper management of SRBDs, which can potentially improve SCI-induced neuropathic pain and cardiovascular dysfunction (see attached his letter of support from SCI Ontario).

Our research plan and budget includes key steps and corresponding financial support for the establishment of an integrated knowledge translation strategy in collaboration with Ms. Xia and Mr. Athanasopoulos, SCI Ontario, healthcare professionals, hospital administrators and policy makers.

- Benefits to the Institution

The linkage of SRBDs with neuropathic pain, cardiovascular dysfunction and spasticity as well as the feasibility of the home-based sleep test will be reported in different means. The knowledge transfer plan includes the following outputs: (i) A newsletter to the SCI community that will be prepared in collaboration with our partners from SCI Ontario; (ii) An original article that will be published in a peer-reviewed journal for dissemination in the scientific and medical community; and (iii) Conference abstracts and presentations that will be submitted to national and international scientific meetings targeting scientists, clinicians, other healthcare professionals, administrators and policy makers (e.g. ASIA, ISCoS). The anticipated outcomes will include: (a) Increase awareness of the SRBDs in the SCI population and among healthcare rehab professionals; (b) Facilitate access of individuals living with SCI to home-based sleep studies; and (c) Improve adherence of individuals living with SCI to diagnosis and treatment of SRBDs.

This project will include the following performance measurements:

(1) Outputs:

(i) A newsletter to the consumers to be disseminated to the Ontario SCI community;

(ii) One manuscript submitted to a peer-reviewed medical journal;

(iii) At least one abstract submitted to a national or international scientific meeting.

(2) Outcomes:

(i) Promoting the awareness of the association of SRBDs with neuropathic pain, cardiovascular dysfunction and spasticity after SCI among healthcare professionals by presenting the study results in conferences;

(ii) Implementation of an outpatient/inpatient service for home-based or hospital-unattended sleep study at Lyndhurst Centre in collaboration with Sunnybrook for individuals with SCI;

(iii) Disseminate the results on potential association of neuropathic pain/cardiovascular dysfunction and SRBDs to the consumers throughout a SCI Ontario newsletter.

2.4 Description of the population to be studied

This research project will include English-speaking adults (18 years of age or older) with subacute or chronic (at least 2 months after injury), cervical/thoracic (injury level at C1 to T6), complete or incomplete (AIS A, B, C or D) SCI who were not previously diagnosed and treated for sleep apnea.

2.5 Compliance with the protocol, ICH GCP and the regulatory requirements

All the investigators involved in this protocol, under the supervision of the principal investigator Dr Julio C. Furlan, have committed themselves to follow the instructions established in the protocol, in compliance with the Good Clinical Practice and the policies established by UHN (e.g., respect of participants privacy and dignity, guidelines to record vital signs, responsibility in scientific research, etc.).

3.0 Study Objectives and Hypothesis

3.1 Primary study objectives:
(1) To evaluate the potential association of neuropathic pain with SRBDs in individuals with SCI;
(2) To study the potential relationship between cardiovascular autonomic dysfunction and SRBDs in individuals living with SCI;
(3) To evaluate the potential association of spasticity with SRBDs after SCI.

3.2 Secondary study objectives:
(1) To confirm that a home-based sleep study is a feasible alternative for diagnosis of SRBDs in tetraplegics and paraplegics;
(2) To compare the costs of a home-based sleep study with the conventional polysomnography in an overnight sleep clinic using cost-minimization analysis.

3.3 Study Hypothesis
(a) Individuals with SCI and untreated SRBDs more frequently develop more severe neuropathic pain;
(b) Individuals with SCI and untreated SRBDs develop more significant cardiovascular dysfunction (i.e., reduced heart rate variation, more frequent episodes of autonomic dysreflexia during sleep);
(c) Individuals with SCI and untreated SRBDs more frequently develop more severe spasticity.

4. Study Design
4.1. Primary and secondary endpoints
Each participant will undergo the following assessments:

4.1.1 Primary Outcome measures:
a) The presence and intensity of neuropathic pain as assessed using the Visual Analog Scale (VAS; range: 0 to 100) and daily amount of analgesics (pregabalin, neurontin, opioids, etc.) [35];

b) The cardiovascular activity during sleep based on the peripheral arterial tone and overall heart rate variability as recorded using the Bittium Faros™ 180 device and Caretaker™ Medical cNIBP monitor device;
c) The number of episodes of autonomic dysreflexia during sleep and their association with apneas and hypopneas by combining the recorded data from the ApneaLink™, Bittium Faros™ 180 device and Caretaker™ Medical cNIBP monitor devices;
d) The degree of spasticity in the upper and lower limbs as assessed using the Modified Ashworth Scale;
e) Serum CRP level.

4.1.2 Secondary Outcome measures:

(i) Practicality of a home-based sleep test based on the experience of the participants as recorded by the research assistant during the sleep studies;
(ii) Economic analysis comparing costs of a home-based sleep test with polysomnography for diagnosis of SRBDs in tetraplegics and paraplegics.
(iii) STOP-Bang questionnaire will be used to screen participants without (score <3) or with symptoms or signs of SRBDs. A higher STOP-Bang score is associated with a greater degree of SRBD. [36-39]

4.2 Description of the type/design of the study

This is 3-year cross-sectional, prospective study.

(1) Individuals with SCI frequently decline to stay overnight to complete formal polysomnography due to their concerns about availability of proper support, funding for attendants and the accessibility of the sleep disorder clinics. Given this, we decided to use a home-based or hospital-unattended sleep test (ResMed ApneaLink Air™) that is a practical, less costly, validated and reliable surrogate for diagnosis of the SRBDs.[32-34] A trained research assistant from Sunnybrook Health Science Centre with expertise on the setting up and use of ResMed ApneaLink™, Bittium Faros™ 180 and Caretaker™ Medical cNIBP monitor devices will be available to assist the participants who have questions or problems with setting up the sleep apnea testing. The process for the technical support for the home based-sleep study will include the following steps:

   (a) The research assistant from Sunnybrook Health Science Centre will receive a notification by phone from the research coordinator from Lyndhurst stating that a new participant will undergo home-based sleep studies. The study participant identifying number (i.e. "study participant ID #...") along with their address and minimal clinical information (only level and severity of spinal cord injury) will be provided.
   (b) The study participant will receive the name and phone number of the research assistant from Sunnybrook Health Science Centre and will be instruct to call them if there are any questions or concerns only during home-based sleep study (12 hours overnight support).
   (c) If the study participant is unable to set up the home-based sleep study devices, they will be offered to receive a visit from the research assistant at their residence. If the study participant agrees, the research assistant will provide technical support at their residence.

If the study participant disagrees and the setting up is not possible, the study participant will be withdrawn from the study.

(d) At discretion and expenses of the research assistant and Dr. Mark Boulos (co-investigator at Sunnybrook Health Science Centre, the home-based sleep study devices can be either send by especial and secure courier (e.g. FEDEX) or picked up by the research assistant at the study participant’s residence on next the day after the test. The study participant must agree with the latter option before the research assistant can drop by their residence. All the costs for either option are included in the price for the service as per the revised inter-institutional agreement.

(e) The research assistant must properly destroy all PHI right after completing of the home-based sleep studies. The only information that should be maintained includes the study participant ID number and data on how many times and the reasons for phone calls and/or visit to provide technical support.

(2) Individuals living with SCI are often on pharmacologic treatment for neuropathic pain and/or spasticity. Given their potential confounding effect, we will collect information on the medicaments taken at the time of the studies in order to properly analyze the results of both studies.

(3) All the laboratory blood tests will be carried out by a company that provides high-quality services using standardized assays and methods across Canada. This will facilitate the comparisons and interpretation of the results from this research project.

4.3 Study Procedure

Table 1. Study Visits

Event of Assessment	Visit 1(Screening Visit)	Visit 2(Assessment after ApneaLinkTM –home sleep test)
Eligibility Screen	X	
STOP-Bang questionnaire	X	
Visual Analog Scale for pain	X	
Enrollment & ApneaLink Sleep Breathing, Bittium Faros^{TM} 180 and Caretaker^{TM} Medical cNIBP Monitoring	X	
Demographics	X	
History of Injury	X	
General Health History	X	
Laboratory blood tests (CRP and CBC)		X
Participants’ experience on the use of a home-based sleep test		X
Modified Ashworth Scale	X	

Visit 1 and 2 will each last approximately an hour.

Those individuals with clinical warning signs for SRBDs will undergo a home-based sleep study using the ApneaLink™ (Picture 1) device to confirm the diagnosis of SRBD (n=30). Based on the results of the home-based sleep study, individuals with SRBD will be grouped into mild (n=10), moderate (n=10) and severe sleep apnea (n=10). Also, individuals without clinical warning signs for SRBD will undergo a home-based sleep study using ApneaLink™ in order to identify 10 study participants without SRBDs ("control cases").

All participants will also undergo cardiovascular evaluation during sleep using the Bittium Faros™ 180 (Picture 3) and Caretaker™ Medical cNIBP monitor (Picture 2) devices to assess sympathetic activity during sleep in all study participants (n=40). All participants will be examined with regards to their degree of spasticity as assessed using the Modified Ashworth Scale. Also, all participants will undergo laboratory blood tests to measure their serum CRP level.

Furthermore, an economic analysis will be undertaken in order to compare the costs of the home-based sleep study with the conventional polysomnography in a sleep clinic. For this purpose, the costs of the home-based sleep test will be estimated based on our experience during this study. The costs of the conventional polysomnography will be obtained from the sleep clinic at Sunnybrook Hospital where Dr. Mark Boulos, co-investigator in this study, works. The professional and technical fees will be derived from the Ontario Health Insurance Plan (OHIP) – Schedule of Benefits, Physician Services.

All participants who are diagnosed with moderate-to-severe SRBDs during the study will be referred to Dr. Mark Boulos or other sleep specialist for consultation and CPAP therapy. Also, study participants whose CRP levels are greater than 10 mg/L (normal range up to 3 mg/L) will be immediately referred to their family physician (or emergency if symptoms or signs of systemic infection) for further investigations and treatment because a CRP greater than 10 mg/L is suggestive of infection or primary inflammatory disease.

4.4 Number of Participants

Seventy individuals will be invited to take part in this study.
Based on the results of the home-based sleep study, individuals with SRBD will be grouped into mild (minimum of 10), moderate (minimum of 10) and severe sleep apnea (minimum of 10). Individuals without clinical warning signs for SRBD (minimum of 10) will undergo a home-based sleep study using ApneaLink™ in order to identify 10 study participants without SRBDs ("control cases").

5.0 Selection of Subjects

5.1 Inclusion Criteria

To take part in this study participant must:

- Have traumatic or nontraumatic (without risk for disease recurrence) cervical/thoracic (injury level at C1 to T6), complete/incomplete (AIS A, B, C or D) SCI who were not previously diagnosed and treated for sleep apnea.
- English-speaking adults 18 years of age or older
- At least 2 months after injury

Untreated sleep-related breathing disorders as an aggravating factor for neuropathic pain, cardiovascular dysfunction and spasticity following spinal cord injury

5.2 Exclusion criteria:

- Patients with a non-traumatic spinal cord disease at risk for disease recurrence during the study (*e.g.*, demyelinating spine diseases such as neuromyelitis optica and multiple sclerosis, spinal cord malignancy); of note, patients with other causes of non-traumatic spinal cord disease are eligible for this study.
- Concomitant significant diseases of the central nervous system causing neurological deficits (e.g., concomitant traumatic brain injury, stroke, dementia, Parkinsonism, multiple sclerosis)
- Preinjury chronic pain (e.g., fibromyalgia)
- Psychiatric disorders that could affect the home-based sleep study using the ApneaLink^{TM} and cardiovascular evaluations.
- Neuromuscular diseases (e.g., myasthenia gravis)
- Current substance misuse
- History of primary hypersomnia (e.g., narcolepsy, idiopathic hypersomnia). Of note, hypersomnia is defined as prolonged sleep periods (i.e., more than 10 hours per night).

5.3 Withdrawal of Subjects

(a) When and how to withdraw participants from the study intervention?

The participant will be able to quit the trial at any moment if he/she withdraws consent, if his/her health status precludes a safe participation in the study.

The investigator may decide to terminate subject’s participation in the study if he/she has not followed study procedures. This study may be stopped at any time by the investigators.

6. Assessment of Safety

All adverse events will be recorded and used to assess participant safety.

6.1 Adverse Events Monitoring

Adverse events (AE) will be collected continually throughout the study. The study clinician is responsible for reviewing and signing off on documentation of events meeting the criteria and definition of an AE or Reportable Event as provided in this protocol. All AEs and Reportable Events will be recorded. Adverse events will be entered in participant’s charts and recorded on the appropriate case report form (CRF) page from the time written informed consent is obtained until completion of the study or until resolution of the reportable event.
Information to be collected includes the nature, date, time of onset, intensity, duration, causality, and outcome of the event. If an AE is assessed by the Investigator as not reasonably attributable to study intervention, its occurrence must also be recorded in the source documents and reported on the CRF.

6.2 Procedure for Serious Adverse Events

In the case of a Serious Adverse Event (SAE) the site lead clinician or designee must immediately (within 1 working day/24 hours of learning of its occurrence) report the SAE by phone or email to

the study project manager. The signed and dated serious adverse event form must be emailed on the provided CRF to the study project manager within 48 hours of the site's knowledge of the SAE. The site lead clinician must attach a photocopy of all examinations carried out and the dates on which these examinations were performed. Care should be taken to ensure that the participant's identity is protected on all copies of source documents provided to the project manager.
A follow-up on any SAE that is fatal or life threatening should be provided within 5 calendar days. The study clinician is accountable to ensure safety of the participants, including referral to a specialist if indicated. Notably, the study clinician must follow up on the outcome of any adverse events (clinical signs, laboratory values or other, etc.) until they return to normal, or stabilization of the participant's condition. This implies that follow-up may continue after the participant has completed the study.

7. Statistics

The baseline characteristics and outcomes measures among the four study groups will be analyzed using the Kruskal-Wallis test (non-parametric data) or ANOVA (parametric data). Study groups will also be compared using Fisher exact test regarding categorical variables. The costs of the home-based sleep study will be compared to the costs of the polysomnography using Mann-Whitney U test. Sample size and feasibility: Using pain (VAS) as the primary outcome measure, an estimated sample size of 10 subjects per group is required to detect a "minimal clinically important difference" of at least 25 mm in the VAS with a minimum power of 90% and a significance of 5% for 2-sided comparison tests.[40]

8. Direct Access to Source Data/Documents

All information collected will be kept confidential. Each participant will be given a unique identifier number and her/his personal information will be stored in a locked filing cabinet. Only the people in charge of the study at TRI will ever see this information. A special alphanumeric code will be used instead of participants name on any documents or computer files that could be seen by other people. This code will be completely independent and unrelated to (not have anything to do with) the participants' name. Video and photographic data will be labelled with the participant IDs.

UHN Guidelines for Data and Privacy Protection will be followed in the study. In all related files, programs, and reports, participant will be referred to be a code that bears no relation to participants' actual name.

The following people may come to the hospital to look at the study records and at participants' health information to check that the information collected for the study is correct and to make sure the study is following proper laws and guidelines:

- Representatives of the University Health Network (UHN) including the UHN Research Ethics Board
- The study sponsor Ontario Neurotrauma Foundation –Rick Hansen Institute

Untreated sleep-related breathing disorders as an aggravating factor for neuropathic pain, cardiovascular dysfunction and spasticity following spinal cord injury

9. Quality Control and Quality Assurance Procedures

The Participant ID should be entered on all CRFs. Completed CRFs must be signed and dated by the Investigator or designee to signify that the recorded data is complete and accurate.

10. Ethics

10.1 Recruitment

Participants will be recruited using posters placed throughout the University Health Network. Inpatients at Toronto Rehab-Lyndhurst Centre will also be recruited using the TRI Central Recruitment Services (CRS) to identify potential participants. Furthermore, potential participants will be identified by the member of the potential subject's clinical team (e.g. medical doctor). The clinical team member and/or CRS Patient Liaison will identify potential candidates based on the subject's eligibility criteria (inclusion/exclusion). The clinical team member or CRS Patient Liaison will ask the eligible subject if he/she is interested to hear about research; if yes, then the they will send a refferal to the research coordinator. In this way, the subject's confidentiality is not breached.

10.2 Consent process

A research team member who does not participate in the participant's care at Toronto Rehab will meet the participant and inform them about the research purpose, the identity of the researcher, the expected duration and nature of participation, the research procedures and foreseeable risks and benefits associated with participation in the study, both verbally and with the written Informed Consent document(s). When the participant agrees to participate in the study, a signed consent form will be obtained and a detailed chart review will be performed to ascertain that the inclusion and exclusion criteria are met.
The participant will be reminded that he/she can withdraw consent at any moment.

10.3 *Review and approval of the study by the REB*

The present study will not be initiated before approval of the REB and obtaining of the Institutional Authorization from UHN.

10.4 Review and approval of any amendment.

In the unlikely event that an immediate hazard threatened the participants, the protocol will be modified, or suspended, before submitting an amendment to the REB.

Any other type of change will be submitted to the approval of the REB before its implementation to the participants.

11. Data Handling and Record Keeping

Participant data will be collected on Case Report Forms (CRFs). Prior to the start of the study, the PI will complete a Delegation of Authority Form showing the signatures and handwritten initials of all individuals who are authorized to perform study tasks, specifically those who have

been authorized to make or change entries in CRFs. The PI or designee will provide completed CRFs for each participant. All required data are to be recorded in the CRFs.
Completed CRFs will be reviewed by the PI or designee to ensure completeness and consistency. An explanation must be documented for any missing data.

Collected data will be stored at hospital network drive. This drive will be backed up on regular basis and will be protected using a firewall. In the event the firewall is not functioning, the hard drives with the participants' data have to be disconnected from Local Area Network (LAN) or Wide Area Network (WAN).

Collected data will be available for statistical analysis according to the methods described in Section 7 Statistics. After the study is completed and the results are published, data will be stored for ten years. After ten years, the data will be destroyed. In the event of inappropriate release of personal health information the following will be done: further release of information will be stopped, any information that can be retrieved, the UHN Privacy Office and REB will be notified, and then further actions may be taken according to recommendations from the UHN Privacy Office and REB.

12. Financing and Insurance

This study will be conducted by the members of the TRI-UHN under the direction of Dr Furlan, and financially supported by Ontario Neurotrauma Foundation –Rick Hansen Institute, as well as by the Ministry of Health of Ontario.

13. Publication Policy

The authors consent to follow these rules regarding the publication process:
1. Papers/abstracts have to be sent to all co-authors at least 10 days before a submission, and all co-authors should be given ample time to comment and edit the papers/abstracts.
2. No paper/abstract will be submitted unless the principal author has verbal or written permission to submit the paper/abstract from every individual co-author.
3. If a paper/abstract is submitted without following the above 2 rules, the principal author will have to pull the paper from the conference/journal.

In addition, all the investigators implied in this protocol and potential co-authors are committed to follow institutional guidelines regarding data accuracy, ethic and publication process. Moreover, the journal/congress where the article/abstract will be submitted have their specific guidelines.

The results of this research project will be presented to target audiences in scientific and medical meetings that are focused on spinal cord injury research and clinical practice (e.g. annual meeting of the International Spinal Cord Society [ISCOS], National Neurotrauma Symposium, National Spinal Cord Injury Conference, annual meeting of the American Spinal Injury Association [ASIA]). These opportunities will offer ample discussion on the results with experts and consumers.

14. References:

1. Noreau L, Proulx P, Gagnon L, et al. Secondary impairments after spinal cord injury: a population-based study. *Am J Phys Med Rehabil* 2000;79(6):526-35.
2. Noreau L, Noonan VK, Cobb J, et al. Spinal cord injury community survey: a national, comprehensive study to portray the lives of canadians with spinal cord injury. *Top Spinal Cord Inj Rehabil* 2014;20(4):249-64. doi: 10.1310/sci2004-249
3. Loh E, Guy SD, Mehta S, et al. The CanPain SCI Clinical Practice Guidelines for Rehabilitation Management of Neuropathic Pain after Spinal Cord: introduction, methodology and recommendation overview. *Spinal Cord* 2016;54 Suppl 1:S1-6. doi: 10.1038/sc.2016.88
4. Bisogni V, Pengo MF, Maiolino G, et al. The sympathetic nervous system and catecholamines metabolism in obstructive sleep apnoea. *J Thorac Dis* 2016;8(2):243-54. doi: 10.3978/j.issn.2072-1439.2015.11.14
5. Rupp T, Peyrard A, Tamisier R, et al. Cerebral and Muscle Oxygenation During Intermittent Hypoxia Exposure in Healthy Humans. *Sleep* 2016;39(6):1197-9. doi: 10.5665/sleep.5830
6. Phadke CP, Balasubramanian CK, Ismail F, et al. Revisiting physiologic and psychologic triggers that increase spasticity. *Am J Phys Med Rehabil* 2013;92(4):357-69.
7. Graham ID, Logan J, Harrison MB, et al. Lost in knowledge translation: time for a map? *J Contin Educ Health Prof* 2006;26(1):13-24. doi: 10.1002/chp.47 [published Online First: 2006/03/25]
